# Supplementary material for: CO2 and CH4 dynamics in a eutrophic tropical Andean reservoir
Source: PLoS One. 2024 Mar 20;19(3):e0298169. doi: 10.1371/journal.pone.0298169 (PMC10954145; doi:10.1371/journal.pone.0298169)
Supplement: S1 Appendix — (PDF) [file pone.0298169.s001.pdf]

S1 Appendix

S1A Appendix. Weather stations in the study

A weather station (Davis Vantage Pro2) was installed floating on the water surface (2.0 m height) near P1 allowed to rotate and a static weather station (WeatherHawk XP/X) was located on the dam. Both stations monitored meteorological variables including wind speed, air temperature, relative humidity, atmospheric pressure and solar radiation with a sampling resolution of 1 h. Data were also available from a fixed meteorological station from the reservoir operator (El Roble) located on land 2.7 km from the reservoir at coordinates 06°57'41.9''N, -75°07'55.7''W. The floating station was taken as the main data source for the analysis due to its ideal location in relation to the flux measurements, and missing data were filled after correlation with data from the other two stations (See table below).

Correlations between meteorological variables measured by different weather stations. Conventions: The floating weather station (F) versus the weather station placed on the dam (D) and the floating station (F) versus the El Roble (R) weather station (data from the different stations are indicated subscripts).

	Floating (F) vs. Dam (D)	Floating (F) vs. El Roble (R)
Air temperature, T_{air} (°C)	$T_{air\_F} = 0.868T_{air\_D} + 3.24$<br>$r^2 = 0.972$	$T_{air\_F} = 0.630T_{air\_R} + 11.1$<br>$r^2 = 0.760$
Wind speed, U (m s^{-1})	$U_F = 0.401U_D + 1.11$<br>$r^2 = 0.763$	$U_F = 1.53U_R + 1.22$<br>$r^2 = 0.314$
Relative humidity, H (%)	$H_F = 32.7H_D^{0.243}$<br>$r^2 = 0.928$	$H_F = 0.756H_R + 20.8$<br>$r^2 = 0.798$

S1B Appendix. Bubbles detection

CH_4 flux measurements that were affected by ebullition were discarded for the analysis of diffusive fluxes, since Eq (2) (main manuscript) is only valid for describing diffusive fluxes [1]. In general, ebullition was not considered for further analysis due to the low representative sampling size of this highly variable phenomenon. To identify bubble-affected chamber deployments, we used the surface renewal model [2] by estimating the highest theoretically possible gas transfer velocity (k_{600_max}) for the wind speed and water temperature during the chamber deployments ($k_{600\_SRM} = A\ 600^{-n}(\epsilon v)^{1/4}$), where A is an empirical coefficient, ϵ is the dissipation rate of the turbulent kinetic energy estimated as a function of the friction velocity produced by wind speed and v is the kinematic viscosity. We estimated that the maximum theoretical dissipation rate ϵ and the maximum kinematic viscosity (v) were 4×10^{-6} $m^3\ s^{-2}$ and 9.15×10^{-7} $m^2\ s^{-1}$, respectively, used the maximum proportionality coefficient reported in the literature (A = 1.46) [3], to be conservative, obtaining k_{600_max} = 30 cm h^{-1} for the

maximum measured wind speed. The following is the equation used for estimating the dissipation rate of the turbulent kinetic energy estimated as a function of the friction velocity:

$$\epsilon_{u*} = u*^3/(\kappa\, z\,);\ u* = \left(\frac{\rho_a C_d U^2}{\rho_w}\right)^{0.5}$$

where u* is the friction velocity computed from shear stress at the air-water interface following [4], κ is the von Karman constant (0.41), z is the water depth of the estimate (0.15 m), ρ_a and ρ_w are the density of air and water, respectively, U is the wind velocity measured 2 m above the water surface and C_d is the drag coefficient considering atmospheric stability following [5].

S1C Appendix. Assumptions for the advective component in the mass balance

The advection was estimated using the main wind speed direction along the main axis of the reservoir as the driver of advective flow at the water surface, where the estimated vertically averaged horizontal velocity at the water surface was ~1 cm s^{-1} (0 to 2.6 cm s^{-1}). Under these conditions, the water travel time from the inflow zone P3, to the mid-lake zone P2, and from P2 to the dam zone P1 (3150 m and 5880 m apart, respectively) was approximately ~3.6 and 6.8 d. These estimates are rather conservative, as the wind direction reversed between morning and afternoon. Therefore, spatial gradients were assumed to have no significant changes during the time elapsed between sampling of neighboring sites (1-2 days). On the other hand, lateral transport was assumed to be negligible because the wind direction was mostly aligned with the longitudinal extent of the reservoir, and because the steep slopes of the canyon-like reservoir are not favorable for lateral transport driven by differential cooling or heating.

S1D Appendix. On the overestimation of the CO_2 concentration

In contrast to the observed negative CO_2 fluxes, the CO_2 concentration in the surface water was consistently higher than the atmospheric equilibrium concentration (~13.0 µmol L-1). We therefore concluded that CO_2 surface concentration measurements were unreliable. It is widely known that

dissolved CO_2 is affected by the dynamic chemical equilibrium with other carbonate species [6], which makes the headspace method potentially flawed [7] and this effect has been typically assumed to be small [8]. However, recent research has proved that the headspace method, used in the gas chromatography procedure, can largely overestimate CO_2 concentrations for under-saturated samples or pH above 7.5, which represent typical conditions in eutrophic systems [7]. It is possible to correct CO_2 concentration estimates using pH and alkalinity, with better approximations in waters with neutral to high pH and high alkalinities (>1000 µmol/L), otherwise, overestimation (between 50 and 300%) can occur [9]. In this study, however, alkalinity was not measured, so the correction could not be applied. For this reason, in future studies, water conditions should be considered before deciding to apply the headspace technique or to use direct in situ pCO_2 measurement methods.

References

1. Bastviken D, Cole J, Pace M, Tranvik L. Methane emissions from lakes: Dependence of lake characteristics, two regional assessments, and a global estimate. Global Biogeochem Cycles. 2004;18: 1–12. doi:10.1029/2004GB002238
2. Lamont JC, Scott DS. An Eddy Cell Model of Mass Transfer into the Surface of a Turbulent Liquid. AIChE Journal. 1970;16: 513–519. doi:10.1002/aic.690160403
3. Esters L, Landwehr S, Sutherland G, Bell TG, Christensen KH, Saltzman ES, et al. Parameterizing air-sea gas transfer velocity with dissipation. J Geophys Res Oceans. 2017;122: 3041–3056. doi:10.1002/2016JC012088
4. MacIntyre S, Romero J, Kling GW. Spatial-temporal variability in surface layer deepening and lateral advection in an embayment of Lake Victoria, East Africa. Limnol Oceanogr. 2002;47: 656–671. doi:10.4319/lo.2002.47.3.0656
5. Verburg P, Antenucci JP. Persistent unstable atmospheric boundary layer enhances sensible and latent heat loss in a tropical great lake: Lake Tanganyika. J Geophys Res. 2010;115: 1–13. doi:10.1029/2009JD012839
6. Stumm W, Morgan JJ. Chemical Equilibria and Rates in Natural Waters. Aquatic chemistry. 1996.
7. Koschorreck M, Prairie YT, Kim J, Marcé R. Technical note : CO2 is not like CH4 – limits of and corrections to the headspace method to analyse pCO 2 in fresh water. Biogeosciences. 2021;18: 1619–1627. doi:10.5194/bg-18-1619-2021
8. Hope D, Dawson JJC, Cresser MS, Billett MF. A method for measuring free CO2 in upland streamwater using headspace analysis. J Hydrol (Amst). 1995;166: 1–14.

9. Abril G, Bouillon S, Darchambeau F, Teodoru CR, Marwick TR, Tamooh F, et al. Technical note: Large overestimation of pCO2 calculated from pH and alkalinity in acidic, organic-rich freshwaters. Biogeosciences. 2015;12: 67–78. doi:10.5194/bg-12-67-2015
