# Supplementary material for: CO2 and CH4 dynamics in a eutrophic tropical Andean reservoir
Source: PLoS One. 2024 Mar 20;19(3):e0298169. doi: 10.1371/journal.pone.0298169 (PMC10954145; doi:10.1371/journal.pone.0298169)
Supplement: S1 Fig — a. Water surface (blue) and air (red) temperature, b. wind speed, c. solar radiation and d. cloud cover fraction. Grey vertical bars mark the time of the field campaigns. (PDF) [file pone.0298169.s002.pdf]

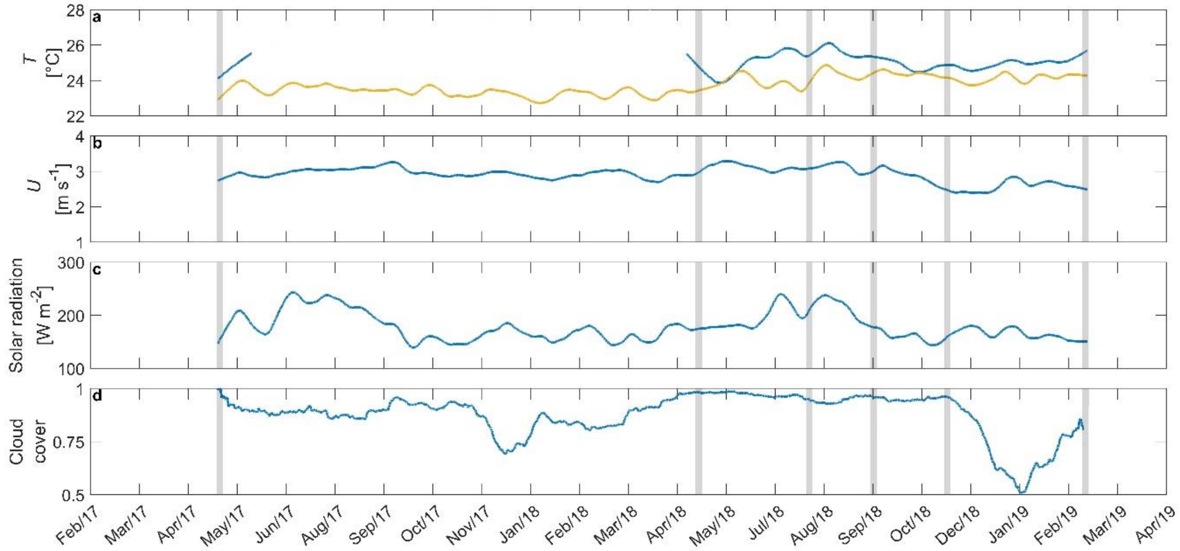

S1 Fig. 30-day-moving average of selected meteorological variables and surface water temperature during the study period (1 h resolution). **a**. Water surface (blue) and air (red) temperature, **b**. wind speed, **c**. solar radiation and **d**. cloud cover fraction. Grey vertical bars mark the time of the field campaigns.
