# Supplementary material for: CO2 and CH4 dynamics in a eutrophic tropical Andean reservoir
Source: PLoS One. 2024 Mar 20;19(3):e0298169. doi: 10.1371/journal.pone.0298169 (PMC10954145; doi:10.1371/journal.pone.0298169)
Supplement: S2 Fig — (PDF) [file pone.0298169.s003.pdf]

S2 Fig Physicochemical profiles and vertical structure of Porce III reservoir

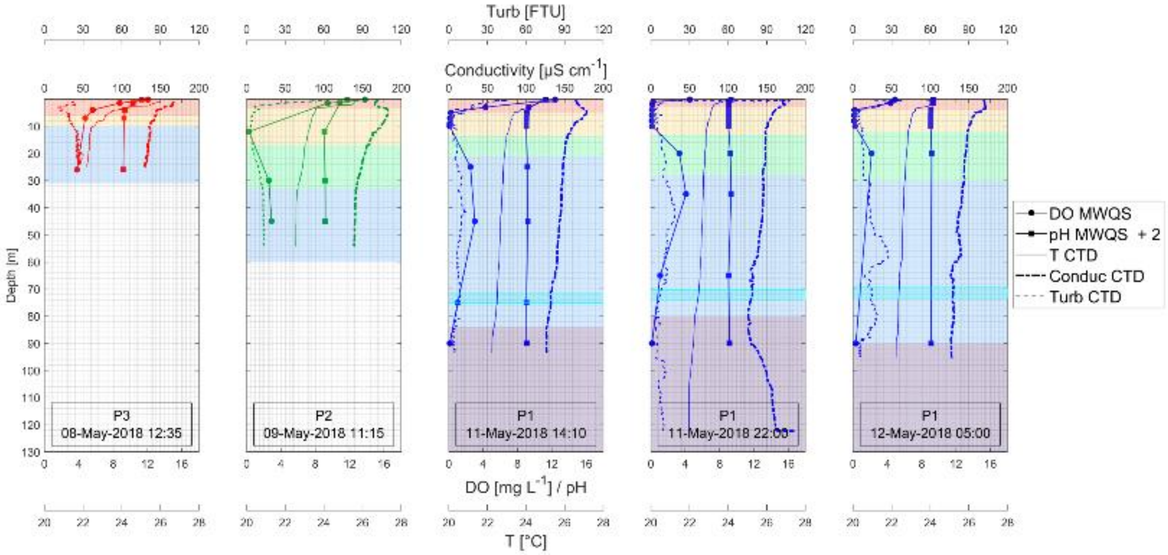

S2A Fig Physicochemical profiles and vertical structure of Porce III reservoir during the high-level-wet campaign C2-H-Wet. Dissolved oxygen (DO), pH, water temperature (T), electrical conductivity (Conduc) and turbidity (Turb) measured with the multiparameter water quality sonde (EXO YSI, MWQS) or the CTD (SeaBird 25). The background color represents the layers according to the previously defined conventions.

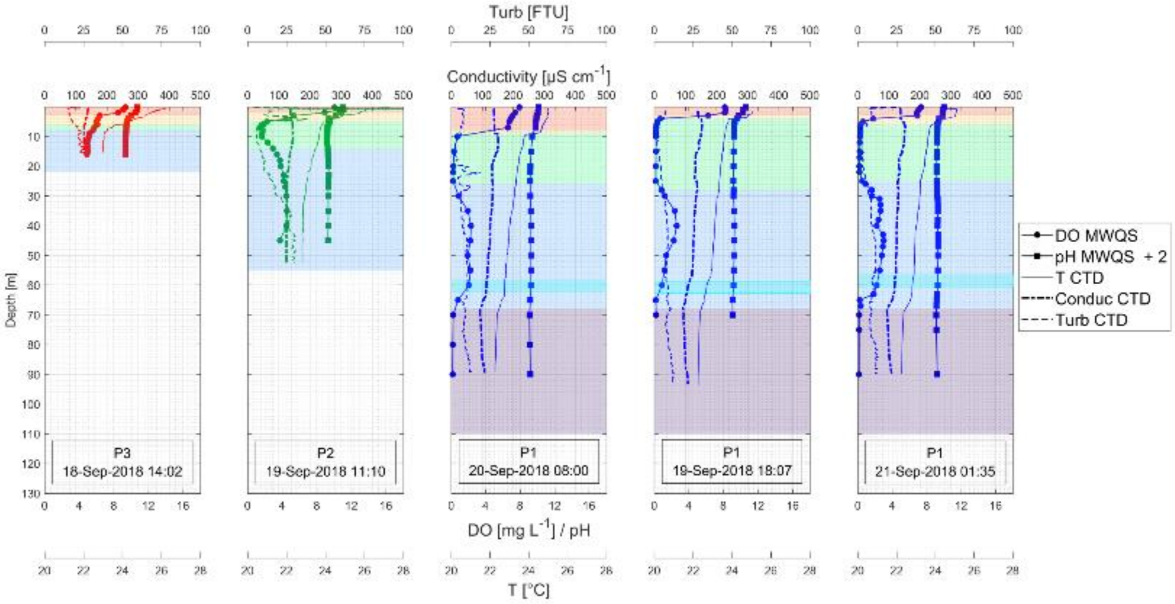

S2B Fig Physicochemical profiles and vertical structure of Porce III reservoir during the low-level-dry-wet-transition campaign C4-L-DWT. Abbreviations and background color code are explained in the S2 Fig caption.

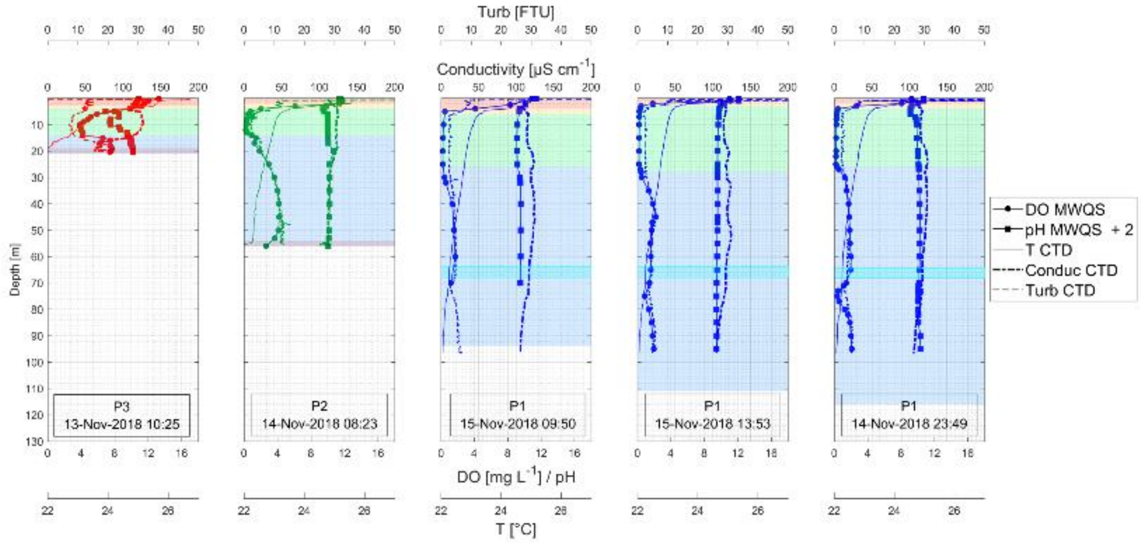

S2C Fig Physicochemical profiles and vertical structure of Porce III reservoir during the medium-level-wet campaign C5-M-Wet. Abbreviations and background color code are explained in the S2 Fig caption.

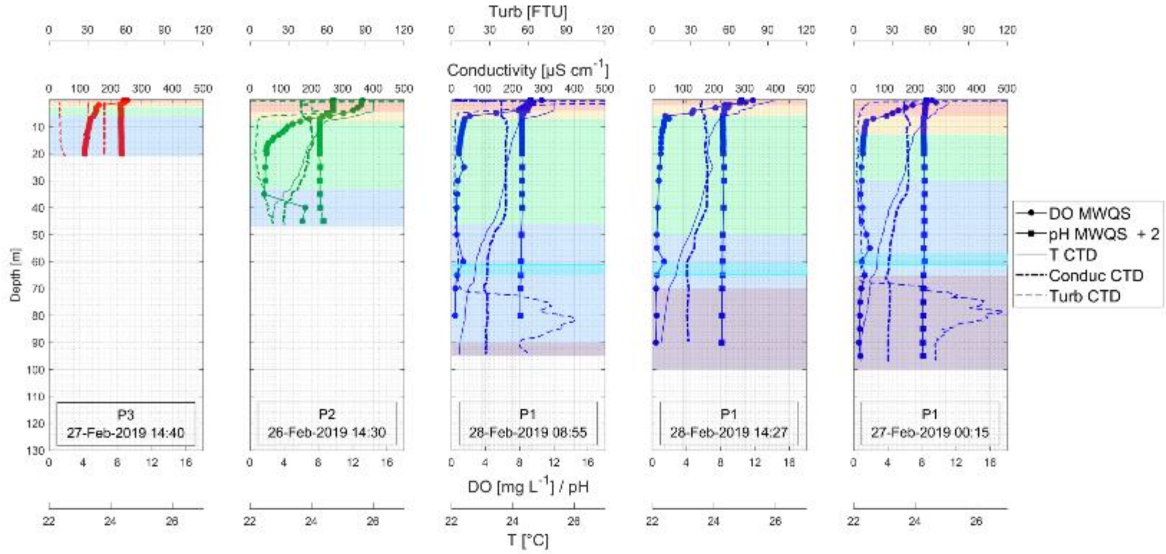

S2D Fig Physicochemical profiles and vertical structure of Porce III reservoir during the medium-level-dry campaign C6-M-Dry. Abbreviations and background color code are explained in the S2 Fig caption.
