# Supplementary material for: CO2 and CH4 dynamics in a eutrophic tropical Andean reservoir
Source: PLoS One. 2024 Mar 20;19(3):e0298169. doi: 10.1371/journal.pone.0298169 (PMC10954145; doi:10.1371/journal.pone.0298169)
Supplement: S3 Fig — The background color represents the layers according to the previously defined conventions (Fig 1B–main manuscript). Hidden values: right panel 399 μmol L-1 at 115 m depth. (PDF) [file pone.0298169.s004.pdf]

S3 Fig. Dissolved CO_2 and CH_4 profiles

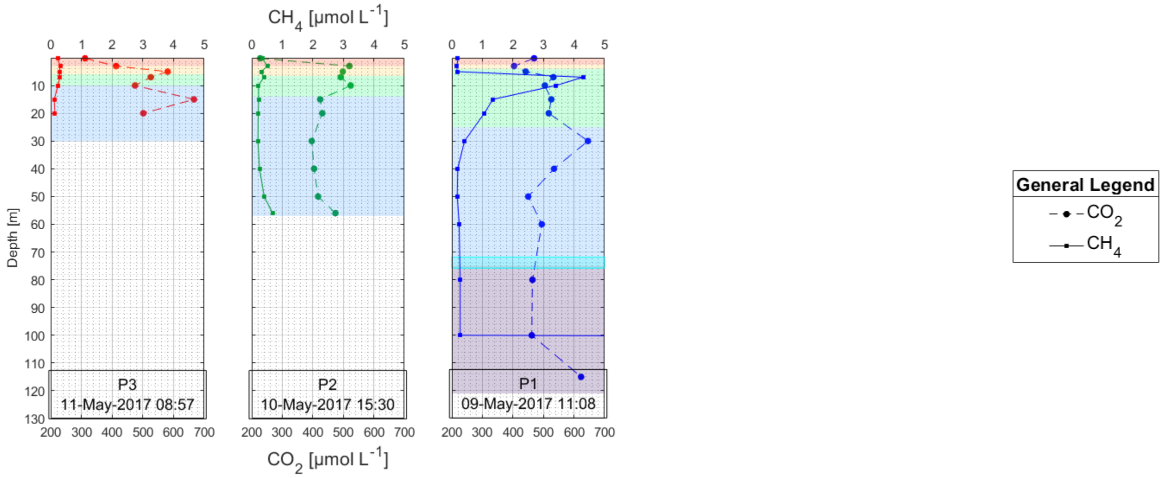

S3A Fig. Dissolved CO_2 and CH_4 profiles during the high-level-wet campaign C1-H-Wet. The background color represents the layers according to the previously defined conventions (Fig. 1b – main manuscript). Hidden values: right panel 399 μmol L^{-1} at 115 m depth.

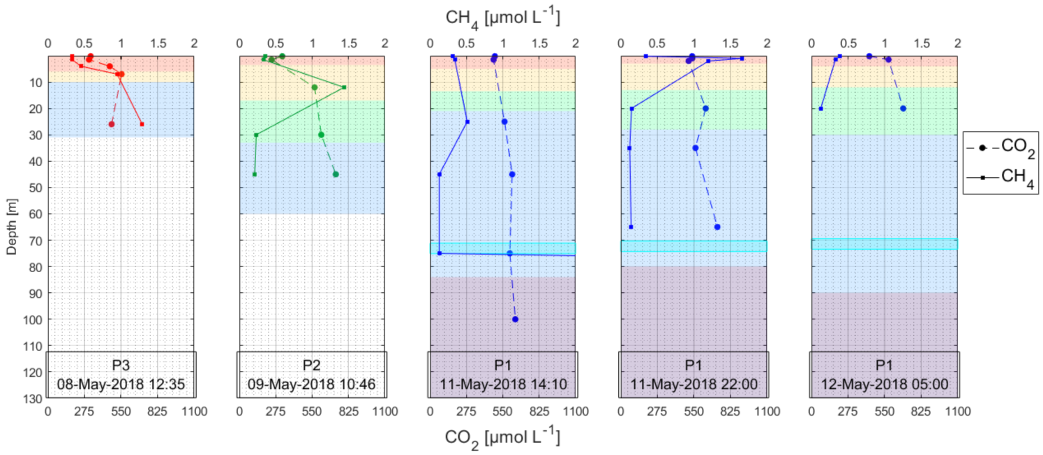

S3B Fig. Dissolved CO_2 and CH_4 profiles during the high-level-wet campaign C2-H-Wet. The background color represents the layers according to the previously defined conventions. Hidden values: third panel left to right 53.3 μmol L^{-1} at 100 m depth.

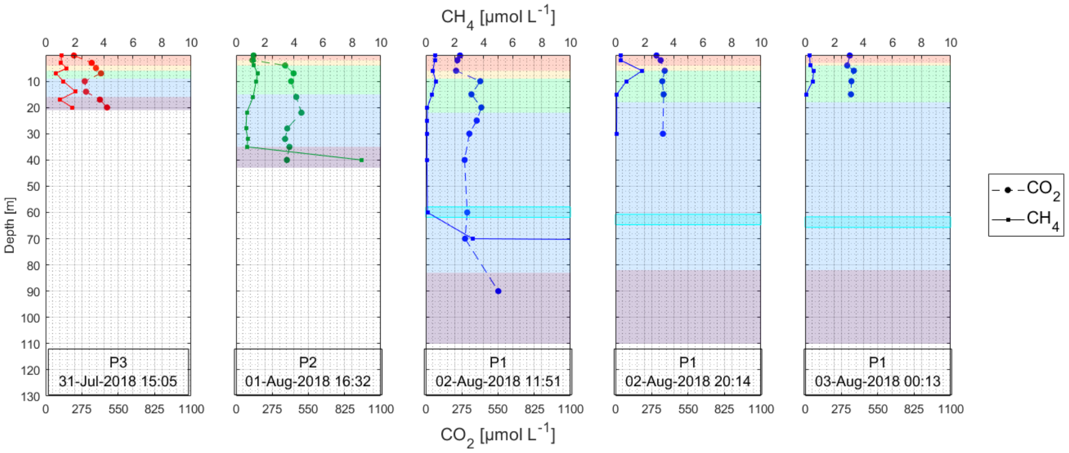

S3C Fig. Dissolved CO_2 and CH_4 profiles during C3-L-Dry. The background color represents the layers according to the previously defined conventions. Hidden values: third panel left to right 474 μmol L^{-1} at 90 m depth.

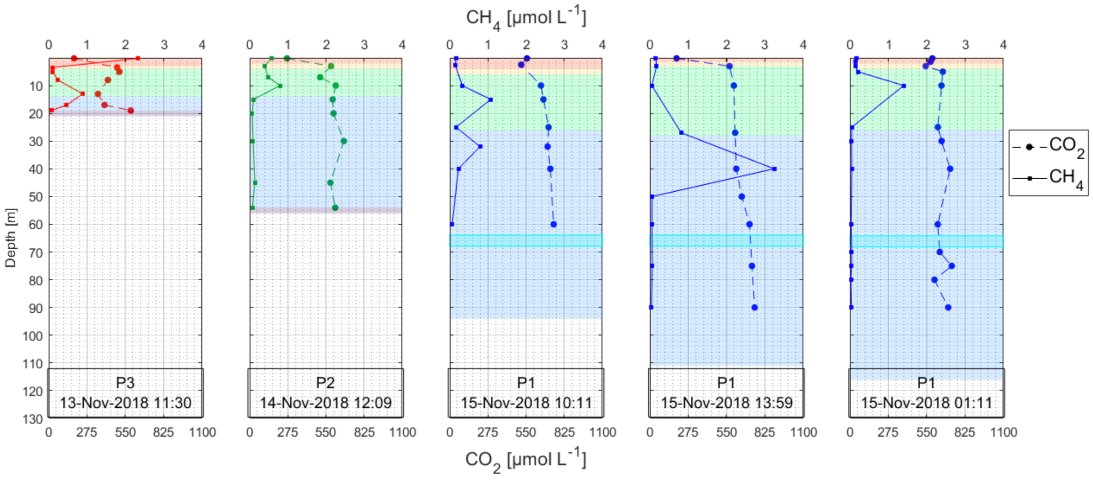

S3D Fig. Dissolved CO_2 and CH_4 profiles during C5-M-Wet for each sampling point. The background color represents the layers according to the previously defined conventions.

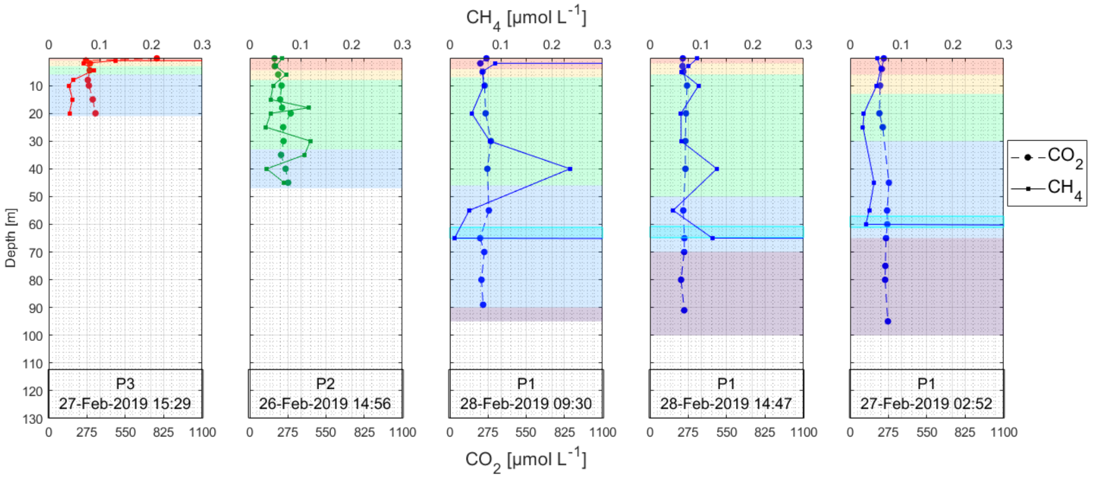

S3E Fig. Dissolved CO_2 and CH_4 profiles during C6-M-Dry. The background color represents the layers according to the previously defined conventions (Fig. 1b – main manuscript). Hidden values: third panel left to right 10.5, 6.73 and 3.384 μmol L^{-1} at 70, 80 and 89 m depth, respectively; forth panel 20.8, 31.2 and 49.8 μmol L^{-1} at 70, 80 and 91 m depth; right panel 20.5 and 20.8 μmol L^{-1} at 80 and 95 m depth, respectively.
