# Supplementary material for: CO2 and CH4 dynamics in a eutrophic tropical Andean reservoir
Source: PLoS One. 2024 Mar 20;19(3):e0298169. doi: 10.1371/journal.pone.0298169 (PMC10954145; doi:10.1371/journal.pone.0298169)
Supplement: S4 Fig — (PDF) [file pone.0298169.s005.pdf]

**S4 Fig. Surface concentration of $CH_4$ and diffusive fluxes and several forcings**

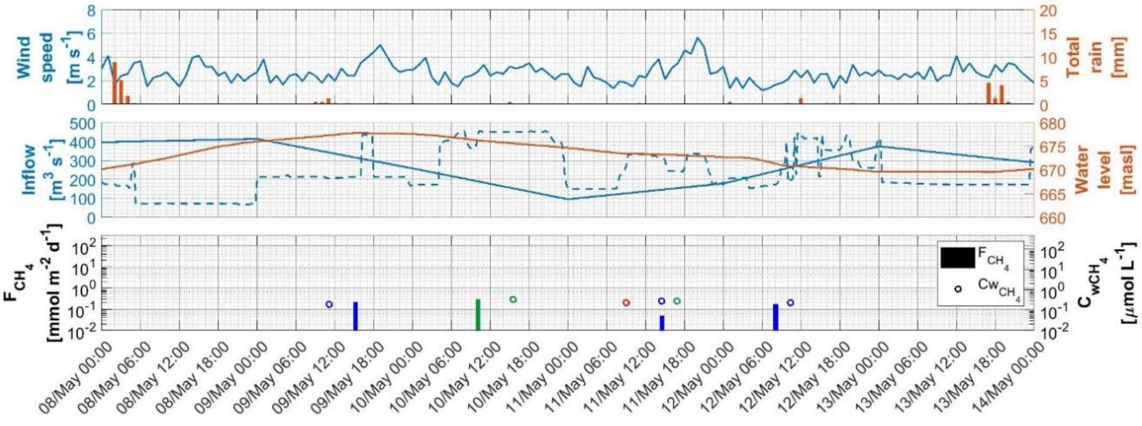


**S4A Fig. Surface concentration of $CH_4$ and diffusive fluxes and several forcings during the high-level-wet campaign of May/2017 C1-H-Wet**. In the middle panel total inflow in blue solid line (1 day of resolution) and the sum of the discharges of the upstream reservoirs Porce II and Troneras in blue dashed line (1 hour of resolution). In the lower panel, colors indicate sampling points: P3 (red), P2 (green), P1 (blue).

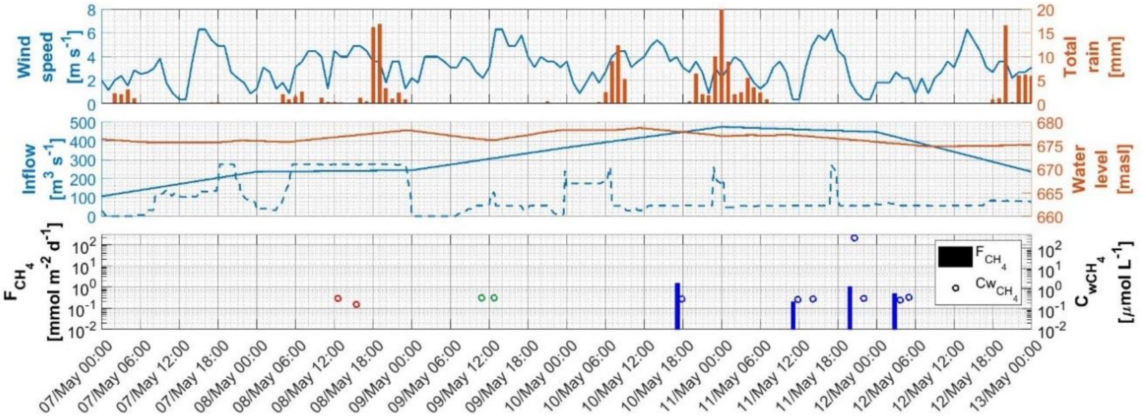


**S4B Fig. All measured surface concentration of $CH_4$ and diffusive fluxes during the high-level-wet campaign of May/2018 C2-H-Wet and the time series of several forcings**. In the middle panel total inflow in blue solid line (1 day of resolution) and the sum of the discharges of the upstream reservoirs Porce II and Troneras in blue dashed line (1 hour of resolution). In the lower panel, colors indicate sampling points: P3 (red), P2 (green), P1 (blue).

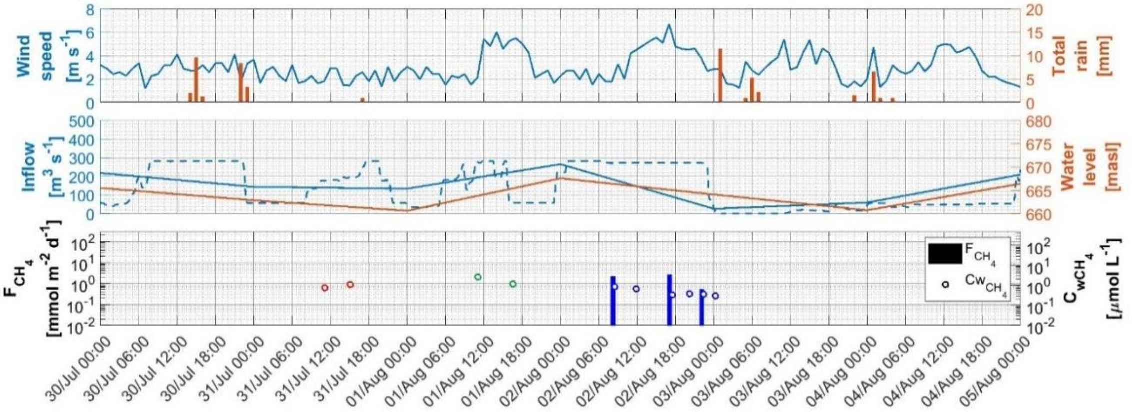


**S4C Fig. All measured surface concentration of $CH_4$ and diffusive fluxes during the high-level-dry campaign of Aug/2018 C3-L-Dry and the time series of several forcings**. In the middle panel total inflow in blue solid line (1 day of resolution) and the sum of the discharges of the upstream reservoirs Porce II and

Troneras in blue dashed line (1 hour of resolution). In the lower panel, colors indicate sampling points: P3 (red), P2 (green), P1 (blue).

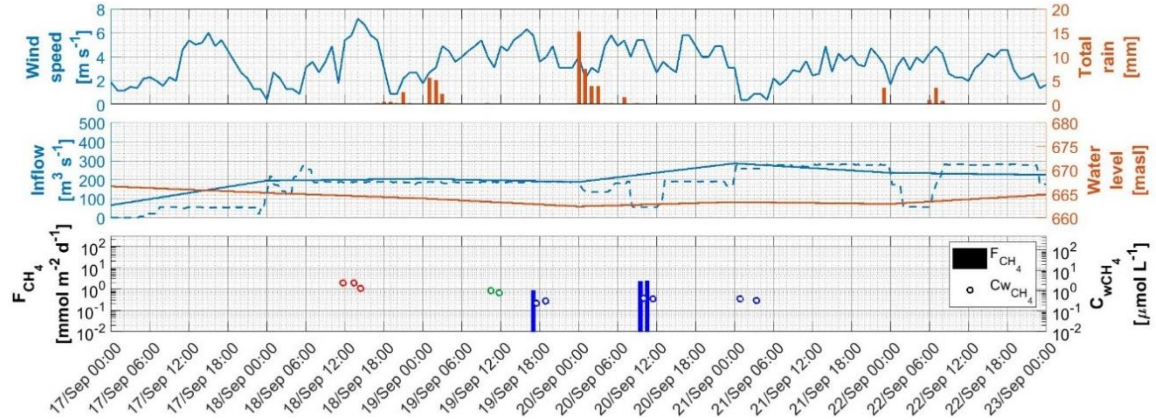


**S4D Fig. All measured surface concentration of $CH_4$ and diffusive fluxes during the low-level-dry-wet campaign of Sep/2018C4-L-DWT and the time series of several forcings**. In the middle panel total inflow in blue solid line (1 day of resolution) and the sum of the discharges of the upstream reservoirs Porce II and Troneras in blue dashed line (1 hour of resolution). In the lower panel, colors indicate sampling points: P3 (red), P2 (green), P1 (blue).

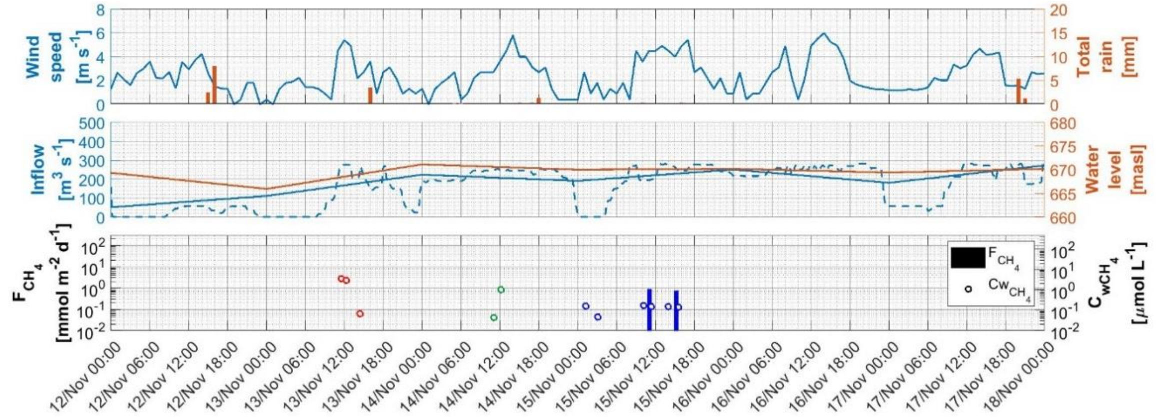


**S4E Fig. All measured surface concentration of $CH_4$ and diffusive fluxes during the medium-level-wet campaign of Nov/2018 C5-M-Wet and the time series of several forcings**. In the middle panel total inflow in blue solid line (1 day of resolution) and the sum of the discharges of the upstream reservoirs Porce II and Troneras in blue dashed line (1 hour of resolution). In the lower panel, colors indicate sampling points: P3 (red), P2 (green), P1 (blue).

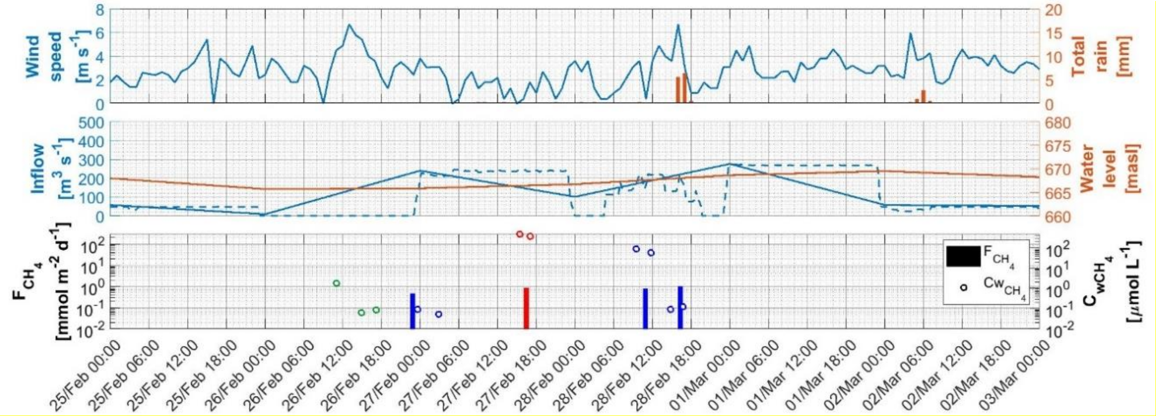


**S4F Fig. All measured surface concentration of $CH_4$ and diffusive fluxes during the medium-level-dry campaign of Feb/2019 C6-M-Dry and the time series of several forcings**. In the middle panel total inflow in

blue solid line (1 day of resolution) and the sum of the discharges of the upstream reservoirs Porce II and Troneras in blue dashed line (1 hour of resolution). In the lower panel, colors indicate sampling points: P3 (red), P2 (green), P1 (blue).
