# Supplementary material for: CO2 and CH4 dynamics in a eutrophic tropical Andean reservoir
Source: PLoS One. 2024 Mar 20;19(3):e0298169. doi: 10.1371/journal.pone.0298169 (PMC10954145; doi:10.1371/journal.pone.0298169)
Supplement: S5 Fig — (PDF) [file pone.0298169.s006.pdf]

S5 Fig. ϵ_m and K_z profiles

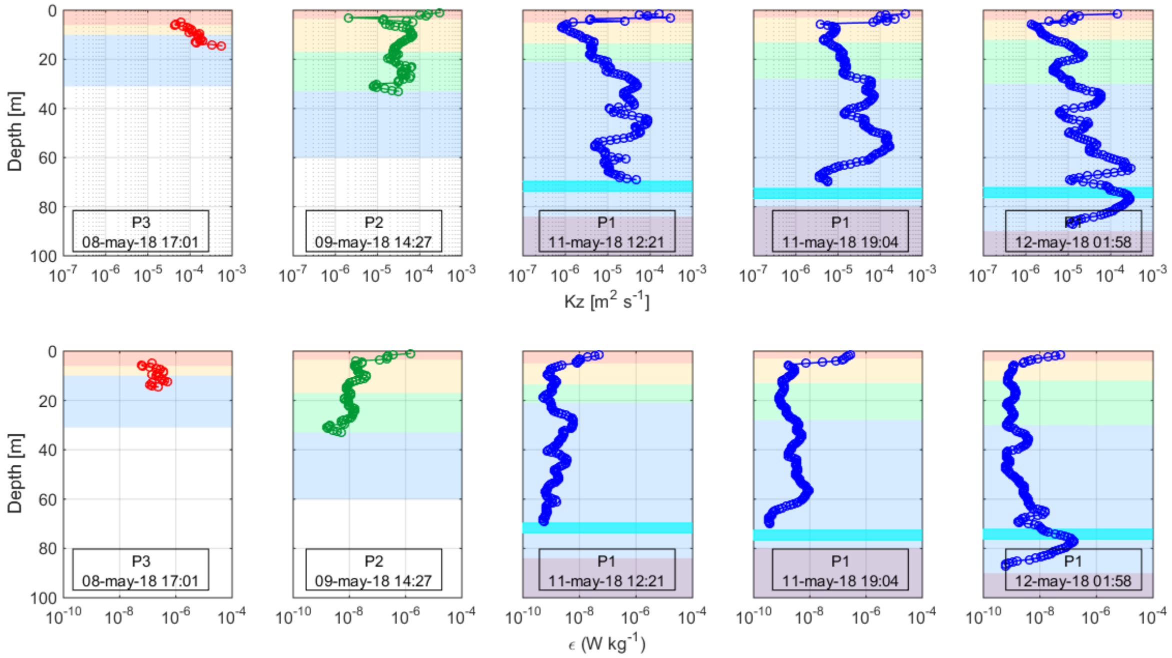

S5A Fig ϵ_m and K_z profiles during the high-level-wet campaign C2-H-Wet The background color represents the layers according to the previously defined conventions.

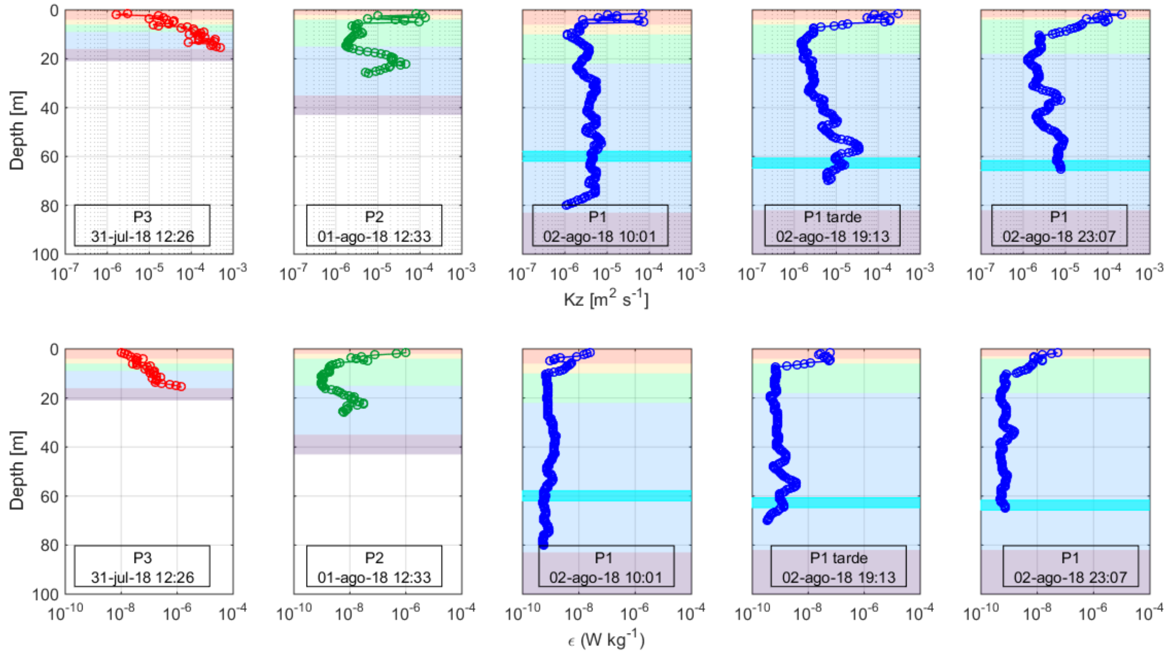

S5B Fig. ϵ_m and K_z profiles during the low-level-Dry campaign C3-L-Dry. The background color represents the layers according to the previously defined conventions (Fig 1b).

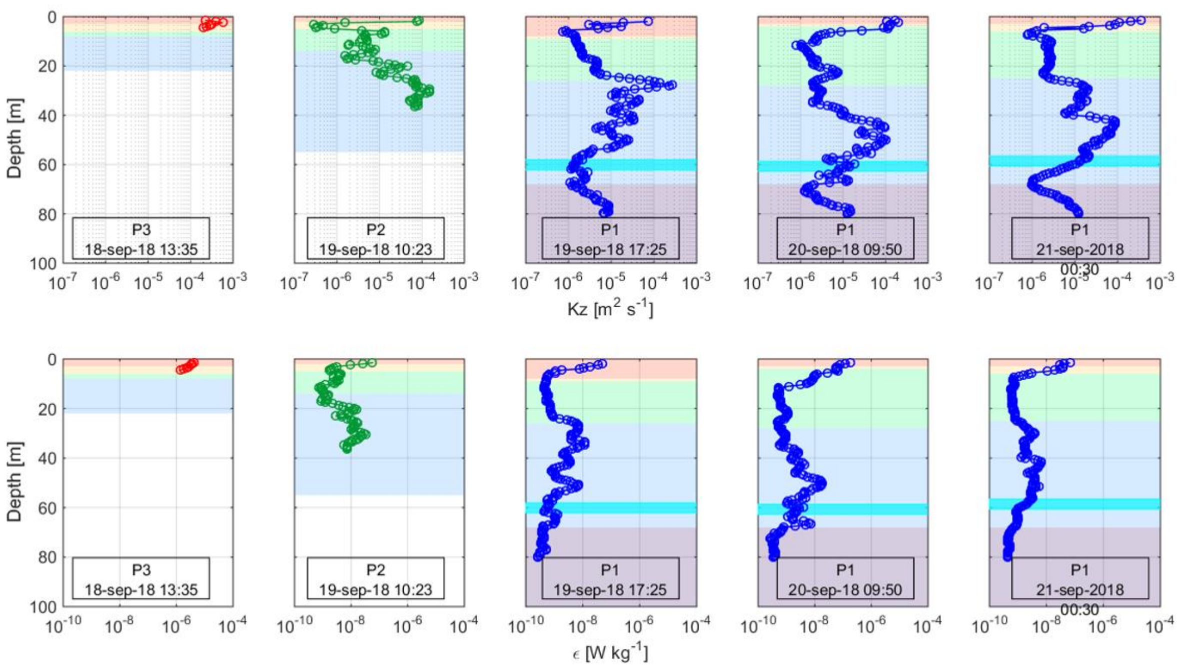

S5C Fig. ϵ_m and K_z profiles during the low-level-dry-wet-transition campaign C4-L-DWT. The background color represents the layers according to the previously defined conventions (Fig 1b).

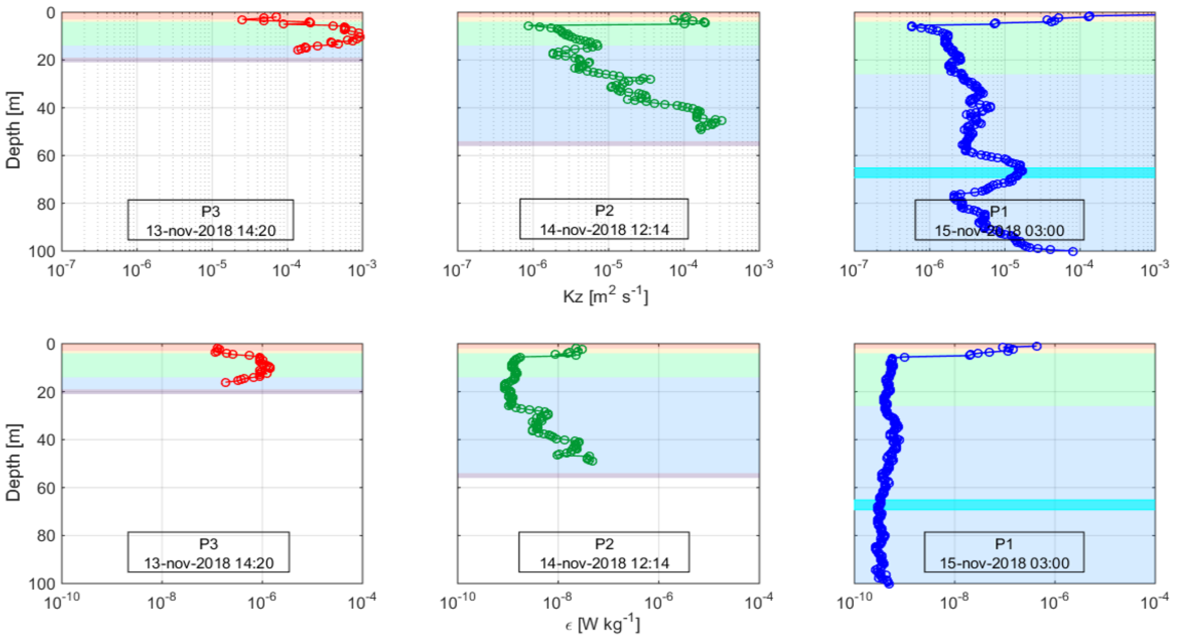

S5D Fig. ϵ_m and K_z profiles during the medium-level-wet campaign C5-M-Wet. The background color represents the layers according to the previously defined conventions (Fig 1b). It was not possible to obtain data during the medium-level-wet campaign C5-M-Wet at the dam zone in the morning and afternoon samplings (P1-M and P1-A), as well as at all sampling points during the medium-level-dry campaign C6-M-Dry due to instrument damage.
