# Supplementary material for: CO2 and CH4 dynamics in a eutrophic tropical Andean reservoir
Source: PLoS One. 2024 Mar 20;19(3):e0298169. doi: 10.1371/journal.pone.0298169 (PMC10954145; doi:10.1371/journal.pone.0298169)
Supplement: S6 Fig — a. high-level-wet C2-H-Wet, b. low-level-dry C3-L-Dry, c. low-level-dry-to-wet-transition C4-L-DWT, d. medium-level-wet C5-M-Wet e. medium-level-dry C6-M-Dry, f. shows a more detailed view on P1-A and P1-N of the final campaign C6-M-Dry. Scales of the subplots are different in order to observe all terms. Positive (negative) bars indicate production (consumption) of CH4 at the SML. (PDF) [file pone.0298169.s007.pdf]

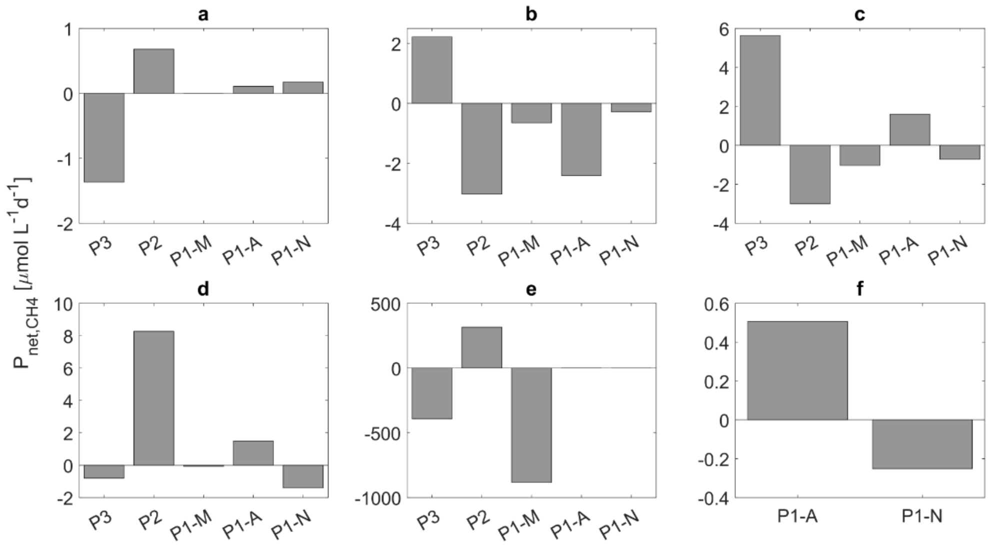

S6 Fig. Estimated net methane production ($P_{net,CH4}$) in the surface. a. high-level-wet C2-H-Wet, b. low-level-dry C3-L-Dry, c. low-level-dry-to-wet-transition C4-L-DWT, d. medium-level-wet C5-M-Wet e. medium-level-dry C6-M-Dry, f. shows a more detailed view on P1-A and P1-N of the final campaign C6-M-Dry. Scales of the subplots are different in order to observe all terms. Positive (negative) bars indicate production (consumption) of CH_4 at the SML.
