# Supplementary material for: CO2 and CH4 dynamics in a eutrophic tropical Andean reservoir
Source: PLoS One. 2024 Mar 20;19(3):e0298169. doi: 10.1371/journal.pone.0298169 (PMC10954145; doi:10.1371/journal.pone.0298169)
Supplement: S7 Fig — Source: Empresas Públicas de Medellín–EPM. (PDF) [file pone.0298169.s008.pdf]

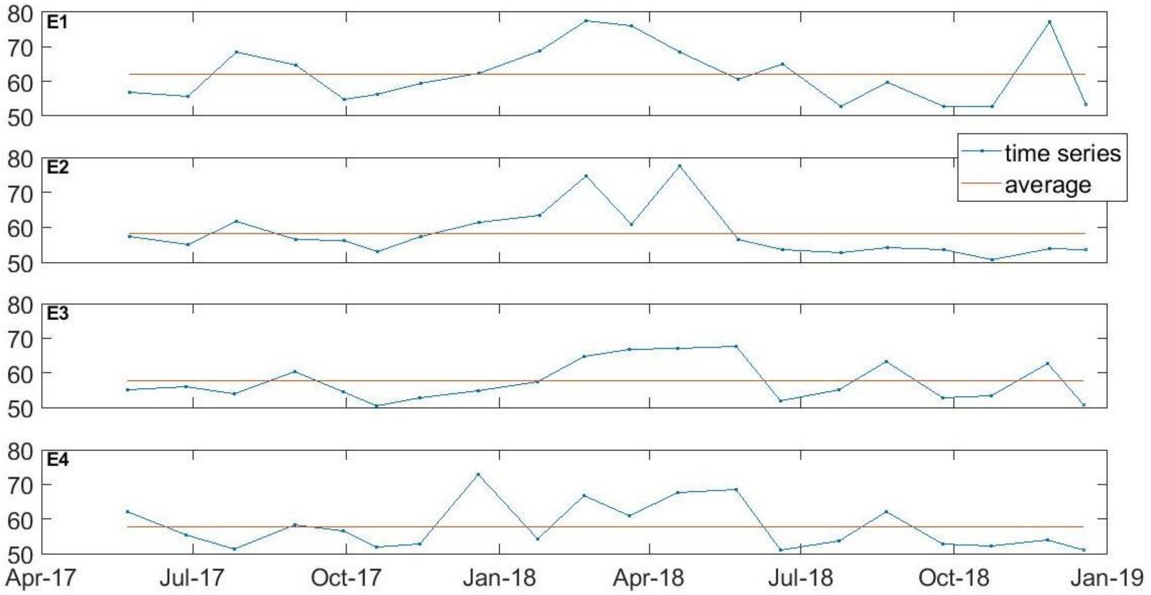

S7 Fig. Trophic state index (TSI). See locations of sampling stations E1 to E4 in Fig 1a (main manuscript). Source: Empresas Públicas de Medellín – EPM.
