# Supplementary material for: CO2 and CH4 dynamics in a eutrophic tropical Andean reservoir
Source: PLoS One. 2024 Mar 20;19(3):e0298169. doi: 10.1371/journal.pone.0298169 (PMC10954145; doi:10.1371/journal.pone.0298169)
Supplement: S1 Table — Surface temperature (T), electrical conductivity (EC), dissolved oxygen (DO), pH, Ammonia (NH3-), nitrites NO2-, nitrates (NO3-), phosphates PO43 and sulfates (SO4-). (PDF) [file pone.0298169.s009.pdf]

**S1 Table. Porce river inflow characteristics during the study period.** Surface temperature (T), electrical conductivity (EC), dissolved oxygen (DO), pH, Ammonia ($NH_3^-$), nitrites $NO_2^-$, nitrates ($NO_3^-$), phosphates $PO_4^3$ and sulfates ($SO_4^-$).

| Date | T [°C] | EC [μS cm$^{-1}$] | DO [% sat] | pH | $NH_3^-$ [mg L$^{-1}$] | $NO_2^-$ [mg L$^{-1}$] | $NO_3^-$ [mg L$^{-1}$] | $PO_4^{3-}$ [mg L$^{-1}$] | $SO_4^{2-}$ [mg L$^{-1}$] |
|---|---|---|---|---|---|---|---|---|---|
| 11-May-2017 | 21.0 | 300 | ND | ND | ND | ND | ND | ND | ND |
| 10-May-2018 | 22.8 | 153 | 54.2 | 7.3 | 1.12 | 0.01 | 1.44 | 0.001 | 283 |
| 31-Jul-2018 | 22.7 | 137 | 55.7 | 7.3 | 1.68 | 0.07 | 2.20 | 0.085 | 10.40 |
| 18-Sep-2018 | 22.6 | 123 | 56.5 | 7.4 | 1.12 | 0.041 | 2.709 | 0.064 | 848 |
| 13-Nov-2018 | 21.2 | 84 | 64.1 | 7.3 | 1.12 | 0.079 | 2.620 | 0.106 | 17.5 |
| 26-Feb-2019 | 25.5 | 70 | 107.2 | 6.9 | 0.84 | 0.018 | 7.92 | 0.021 | 6.23 |
