# Supplementary material for: CO2 and CH4 dynamics in a eutrophic tropical Andean reservoir
Source: PLoS One. 2024 Mar 20;19(3):e0298169. doi: 10.1371/journal.pone.0298169 (PMC10954145; doi:10.1371/journal.pone.0298169)
Supplement: S2 Table — The ID campaign combines a campaign number (C1 to C6), water level condition (high level “H”, low level “L”, medium level “M”) and the seasonal hydrological condition (Wet season “Wet”, Dry-wet transition “DWT”, Dry season “Dry”). (PDF) [file pone.0298169.s010.pdf]

S2 Table. General description of the field campaigns. The ID campaign combines a campaign number (C1 to C6), water level condition (high level "H", low level "L", medium level "M") and the seasonal hydrological condition (Wet season "Wet", Dry-wet transition "DWT", Dry season "Dry").

ID	Date	Water level	Basin's hydrological condition[a]
C1-H-Wet	09 to 12 May/2017	High	Wet season
C2-H-Wet	08 to 12 May/2018	High	Wet season
C3-L-Dry	31 July to 02 Aug/2018	Low	Dry season
C4-L-DWT	18 to 21 Sep/2018	Low	Dry-wet transition
C5-M-Wet	13 to 15 Nov/2018	Medium	Wet season mitigated by the influence of a weak El Niño
C6-M-Dry	26 Feb to 01 Mar/2019	Medium	Dry season, accentuated by El Niño

[a]Based on the monthly reports from the Institute of Hydrology, Meteorology and Environmental studies of Colombia -IDEAM)
