# Supplementary material for: CO2 and CH4 dynamics in a eutrophic tropical Andean reservoir
Source: PLoS One. 2024 Mar 20;19(3):e0298169. doi: 10.1371/journal.pone.0298169 (PMC10954145; doi:10.1371/journal.pone.0298169)
Supplement: S3 Table — (PDF) [file pone.0298169.s011.pdf]

S3 Table. Mean dissipation rates of turbulent kinetic energy (ϵ) and turbulent diffusivity (K_z) in the layers: surface mixed layer (SML), Thermocline (Therm.), Over-plume layer (OPL), river plume (Plume) and Bottom.

Station	Layer	C2-H-Wet		C3-L-Dry		C4-L-DWT		C5-M-Wet		C6-M-Dry	
		ϵ ($m^2 s^{-3}$)	K_z ($m^2 s^{-1}$)	ϵ ($m^2 s^{-3}$)	K_z ($m^2 s^{-1}$)	ϵ ($m^2 s^{-3}$)	K_z ($m^2 s^{-1}$)	ϵ ($m^2 s^{-3}$)	K_z ($m^2 s^{-1}$)	ϵ ($m^2 s^{-3}$)	K_z ($m^2 s^{-1}$)
P3	SML	6.5×10^{-8}	4.5×10^{-5}	1.9×10^{-8}	1.3×10^{-5}	3.3×10^{-6}	3.5×10^{-4}	1.3×10^{-7}	4.9×10^{-5}		
	Therm.	1.9×10^{-7}	9.6×10^{-5}	3.5×10^{-8}	2.8×10^{-5}	2.0×10^{-6}	2.6×10^{-4}	1.3×10^{-7}	4.9×10^{-5}		4.9×10^{-5}
	OPL			8.5×10^{-8}	8.1×10^{-5}			9.1×10^{-7}	5.8×10^{-4}		
	Plume	2.5×10^{-7}	1.7×10^{-4}	1.8×10^{-7}	2.1×10^{-4}			3.9×10^{-7}	1.7×10^{-4}		
	Bottom										
P2	SML	2.3×10^{-7}	1.2×10^{-4}	7.3×10^{-7}	9.6×10^{-5}	4.2×10^{-8}	8.1×10^{-5}				
	Therm.	1.7×10^{-8}	3.8×10^{-5}	2.6×10^{-8}	2.6×10^{-5}	2.4×10^{-9}	4.8×10^{-7}	2.3×10^{-8}	1.1×10^{-4}		1.1×10^{-4}
	OPL	9.3×10^{-9}	3.1×10^{-5}	1.8×10^{-9}	2.8×10^{-6}	2.2×10^{-9}	4.8×10^{-6}	1.4×10^{-9}	3.2×10^{-6}		
	Plume			7.8×10^{-9}	1.5×10^{-5}	8.5×10^{-9}	4.8×10^{-5}	3.9×10^{-9}	1.8×10^{-5}		
	Bottom										
P1 – M	SML	1.0×10^{-8}	4.1×10^{-5}	5.2×10^{-9}	1.3×10^{-5}	9.7×10^{-8}	1.5×10^{-4}				
	Therm.	1.2×10^{-9}	1.6×10^{-6}	3.1×10^{-9}	2.2×10^{-6}	5.9×10^{-8}	1.2×10^{-4}		1.5×10^{-6}*		1.5×10^{-6}*
	OPL	9.6×10^{-10}	4.2×10^{-6}	7.7×10^{-10}	2.7×10^{-6}	7.5×10^{-10}	2.3×10^{-6}				
	Plume	1.5×10^{-9}	2.2×10^{-5}	7.9×10^{-10}	4.5×10^{-6}	2.2×10^{-9}	1.1×10^{-5}				
	Bottom					3.6×10^{-10}	2.5×10^{-6}				
P1 – A	SML	2.2×10^{-7}	1.4×10^{-4}	4.1×10-8	1.1×10^{-4}	1.1×10^{-9}	2.9×10^{-6}				
	Therm.	2.2×10^{-9}	7.4×10^{-6}	4.6×10-8	1.4×10^{-4}	5.3×10^{-10}	1.5×10^{-6}		5.0×10^{-5}*		5.0×10^{-5}*
	OPL	1.2×10^{-9}	1.3×10^{-5}	6.8×10^{-10}	2.0×10^{-6}	6.1×10^{-10}	3.8×10^{-6}				
	Plume	3.4×10^{-9}	5.1×10^{-5}	8.1×10^{-10}	5.8×10^{-6}	2.2×10^{-9}	1.1×10^{-5}				
	Bottom					3.8×10^{-10}	4.5×10^{-6}				
P1 – N	SML	1.4×10^{-8}	1.3×10^{-5}	2.4×10^{-8}	8.4×10^{-5}	4.0×10^{-8}	1.5×10^{-4}	1.2×10^{-7}	1.3×10^{-4}		
	Therm.	1.0×10^{-9}	2.5×10^{-6}	7.9×10^{-9}	5.7×10^{-5}	1.9×10^{-8}	1.6×10^{-5}	1.1×10^{-7}	4.5×10^{-5}		4.5×10^{-5}**
	OPL	9.9×10^{-10}	8.9×10^{-6}	7.6×10^{-10}	2.6×10^{-6}	6.7×10^{-10}	2.4×10^{-6}	4.9×10^{-10}	1.9×10^{-6}		
	Plume	2.9×10^{-9}	4.0×10^{-5}	6.0×10^{-10}	3.9×10^{-6}	2.2×10^{-9}	1.5×10^{-5}	3.9×10^{-10}	4.7×10^{-6}		
	Bottom					4.7×10^{-10}	5.3×10^{-6}				

Unsuccessful measurements of K_z were replaced by averaging the successful measurements (same station and layer) (*) or a repetition of the last measurement (few hours of difference) (**). Empty cells are non-available data.
