# Supplementary material for: CO2 and CH4 dynamics in a eutrophic tropical Andean reservoir
Source: PLoS One. 2024 Mar 20;19(3):e0298169. doi: 10.1371/journal.pone.0298169 (PMC10954145; doi:10.1371/journal.pone.0298169)
Supplement: S4 Table — Water surface temperature during chamber deployments (T), atmospheric fluxes of CO2 (FCO2), atmospheric fluxes of CH4 (FCH4), water surface concentration of CO2 (Cw_CO2), water surface concentration of CH4 (Cw_CH4) and the gas transfer velocity estimated from chambers (k600_CH4). N.A. are not accepted data after quality control. (PDF) [file pone.0298169.s012.pdf]

S4 Table. Atmospheric fluxes and surface concentrations of CO_2 and CH_4 observed during chamber measurements and estimated gas transfer velocities. Water surface temperature during chamber deployments (T), atmospheric fluxes of CO_2 (F_{CO2}), atmospheric fluxes of CH_4 (F_{CH4}), water surface concentration of CO_2 (C_{w_CO2}), water surface concentration of CH_4 (C_{w_CH4}) and the gas transfer velocity estimated from chambers (k_{600_CH4}). N.A. are not accepted data after quality control.

	Time	Station	T [°C]	F_{CO2} [mmol m^{-2} d^{-1}]	C_{w_CO2} * [µmol L^{-1}]	F_{CH4} [mmol m^{-2} d^{-1}]	C_{w_CH4} [µmol L^{-1}]	k_{600_CH4} [cm h^{-1}]
C1-Wet-HW	15:13	P1	25.0	11.1	469	0.20	0.20	3.67
	10:05	P2	27.1	45.0	410	0.26	0.26	3.51
	11:22	P3	24.8	N.A.	312	N.A.	0.23	N.A.
	14:30	P1	24.0	-12.8	119	0.05	0.26	0.68
	16:07	P2	25.6	-33.9	337	N.A.	0.34	N.A.
	8:05	P1	24.2	N.A.	444	0.16	0.24	2.49
C2-Wet-HW	14:53	P3	26.4	-53.0	757	N.A.	0.17	N.A.
	12:15	P2	26.2	N.A.	342	N.A.	0.37	N.A.
	17:10	P1	25.2	15.1	407	1.37	0.32	16.05
	11:05	P1	25.1	-25.7	519	0.19	2.42	0.28
	19:50	P1	24.4	37.0	1006	1.00	336.83	0.01
	2:48	P1	24.6	145.8	670	0.45	0.27	6.10
C3-Dry-DW	10:29	P3	25.3	-15.2	259	N.A.	0.74	N.A.
	10:46	P2	26.1	-26.5	44	N.A.	2.64	N.A.
	12:06	P2	25.5	-36.1	44	N.A.	2.64	N.A.
	8:13	P1	24.6	8.8	118	2.01	0.86	8.59
	17:04	P1	24.6	36.3	277	2.36	0.33	27.51
	22:05	P1	23.6	320.6	369	0.50	0.35	5.52
C4-DWT-DW	11:18	P3	28.1	N.A.	282	N.A.	1.63	N.A.
	13:05	P3	25.4	-9.8	249	N.A.	2.43	N.A.
	10:10	P2	26.7	-27.6	251	N.A.	1.02	N.A.
	17:00	P1	26.5	-4.8	47	0.80	0.25	11.71
	9:28	P1	25.3	67.8	365	2.10	0.42	18.71
	10:33	P1	25.3	26.7	168	2.31	0.42	20.61
	0:25	P1	25.1	76.6	515	N.A.	0.42	N.A.
C5-Wet-RW	11:59	P3	26.6	-18.3	277	N.A.	3.02	N.A.
	14:07	P3	26.3	-25.9	516	N.A.	0.07	N.A.
	9:33	P2	26.3	8.3	538	N.A.	0.04	N.A.
	11:35	P2	26.3	-29.0	412	N.A.	0.04	N.A.
	2:42	P1	24.6	ND	573	N.A.	0.04	N.A.
	11:02	P1	25.3	-30.2	569	0.81	0.15	20.22
	15:11	P1	25.2	-15.8	619	0.69	0.14	18.03
C6-Dry-LW	10:35	P2	26.4	-32.4	240	N.A.	0.58	N.A.
	16:26	P2	26.0	-16.7	228	N.A.	0.09	N.A.
	22:49	P1	26.0	26.9	257	0.44	0.09	16.98
	16:28	P3	25.0	-40.0	956	0.80	369.74	0.01
	10:55	P1	27.8	N.A.	217	0.73	69.27	0.03
	16:20	P1	25.3	N.A.	229	0.91	0.13	27.68

*Dissolved CO_2 concentrations are likely overestimated
